# QTL mapping and BSR-seq revealed loci and candidate genes associated with the sporadic multifoliolate phenotype in soybean (*Glycine max*)

**DOI:** 10.1007/s00122-024-04765-z

**Published:** 2024-11-08

**Authors:** Zhili Wang, Yongchao Niu, Yichun Xie, Cheng Huang, Wai-Shing Yung, Man-Wah Li, Fuk-Ling Wong, Hon-Ming Lam

**Affiliations:** 1 Centre for Soybean Research of the State Key Laboratory of Agrobiotechnology and School of Life Sciences, The Chinese University of Hong Kong, Hong Kong Special Administrative Region, Hong Kong, China; 2 Shenzhen Research Institute, The Chinese University of Hong Kong, Shenzhen, 518057 China; 3 Key Laboratory of the Ministry of Education for Crop Physiology and Molecular Biology, College of Agronomy, Hunan Agricultural University, Changsha, 410128 China

**Keywords:** Multifoliolate, Soybean, BSR-seq, QTL mapping, Differentially expressed gene (DEG)

## Abstract

**Key message:**

The QTLs and candidate genes governing the multifoliolate phenotype were identified by combining linkage mapping with BSR-seq, revealing a possible interplay between genetics and the environment in soybean leaf development.

**Abstract:**

Soybean, as a legume, is typified by trifoliolate leaves. Although multifoliolate leaves (compound leaves with more than three leaflets each) have been reported in soybean, including sporadic appearances in the first compound leaves in a recombinant inbred line (RIL) population from a cross between cultivated soybean C08 and wild soybean W05 from this study, the genetic basis of this phenomenon is still unclear. Here, we integrated quantitative trait locus (QTL) mapping with bulked segregant RNA sequencing (BSR-seq) to identify the genetic loci associated with the multifoliolate phenotype in soybean. Using linkage mapping, ten QTLs related to the multifoliolate trait were identified. Among these, a significant and major QTL, *qMF-2-1* on chromosome 2 and consistently detected across biological replicates, explained more than 10% of the phenotypic variation. Together with BSR-seq analyses, which analyzed the RILs with the highest multifoliolate frequencies and those with the lowest frequencies as two distinct bulks, two candidate genes were identified: *Glyma.06G204300* encoding the transcription factor TCP5, and *Glyma.06G204400* encoding LONGIFOLIA 2 (LNG2). Transcriptome analyses revealed that stress-responsive genes were significantly differentially expressed between high-multifoliolate occurrence lines and low occurrence ones, indicating environmental factors probably influence the appearance of multifoliolate leaves in soybean through stress-responsive genes. Hence, this study offers new insights into the genetic mechanism behind the multifoliolate phenotype in soybean.

**Supplementary Information:**

The online version contains supplementary material available at 10.1007/s00122-024-04765-z.

## Introduction

For plants, leaves are the most vital organs, responsible for producing organic compounds for plant development through capturing light energy in photosynthesis. Generally, leaves can be categorized into two types according to the leaf initiation: simple and compound leaves. A simple leaf contains a single undivided blade, while a compound leaf has multiple leaflets.

Soybeans are one of the most important cash crops in the world, providing abundant oil and protein for humans and livestock as well as supplying industries with raw materials. As a legume, soybean typically has trifoliolate compound leaves, but occasionally some nodes produce more than three leaflets, displaying a multifoliolate phenotype. Artificial and spontaneous multifoliolate soybean mutants with quadri-, quinque-, or even septi-foliolate leaves have been discovered and reported for decades (Fehr 1972; Wang et al. 2001, 2007). Multifoliolate mutants have also been found in other legumes, such as mung bean and alfalfa (Juan et al. 1993a; Soehendi et al. 2007). Compared to the typical trifoliolate phenotype, a multifoliolate leaf possesses a greater surface area and therefore an increased photosynthetic rate, leading to higher yield (Juan et al. 1993b; Soehendi et al. 2007; Zong et al. 2010). In alfalfa, multifoliolate genotypes contain higher protein and essential amino acid contents than trifoliolate varieties (Yancheva et al. 2012). Therefore, understanding the genetic basis of the multifoliolate phenotype is advantageous for improving crop yield and quality.

The exploration of the genetic determinant of the multifoliolate phenotype in soybean can be traced back to the last century. Fehr (1972) discovered that the quinquefoliolate phenotype was linked to the incompletely dominant *Lf1* allele, and the septifoliolate phenotype was determined by the recessive *lf2* allele. Later, two other incompletely dominant alleles, *Lf4* and *Lf5*, were also found to associate with the quinquefoliolate phenotype (Wang et al. 2001). Recently, by delimiting the genetic region of *Lf1*, an AP2/ERF domain-related gene was identified as the candidate gene associated with the soybean multifoliolate trait (Jeong et al. 2017). However, there have been a limited number of genetic studies on the multifoliolate phenotype in soybean development.

Bulked segregant analysis (BSA) is a genetic tool that utilizes two bulked DNA sample pools to rapidly and efficiently map markers linked to genomic regions associated with the trait of interest (Majeed et al. 2022). Combining BSA with transcriptomic data, bulked segregant RNA sequencing (BSR-seq) was developed to take advantage of the analytical powers of both BSA and RNA-seq. By applying BSA to the analysis of bulked RNA sample pools, researchers can not only identify the differentially expressed genes (DEGs), but can also pinpoint single-nucleotide polymorphisms (SNPs) in the transcribed genome between two sample pools (Ramirez-Gonzalez et al. 2015; Gao et al. 2022). Recently, BSR-seq has been widely used to map SNP markers and identify candidate genes for the traits of interest in diverse species (Du et al. 2017; Zhan et al. 2021; Huang et al. 2024).

In the past research, we have frequently come across the multifoliolate phenotype in both wild and cultivated soybeans, most often appearing in the basal node under both field and greenhouse conditions. To gain deeper insights into the genetic basis of the multifoliolate phenotype in soybean, we integrated quantitative trait locus (QTL) mapping with BSR-seq and isolated the associated QTLs in a biparental recombinant inbred line (RIL) population of 407 lines derived from a cross between soybean cultivar C08 and wild soybean W05. Genes residing in the delimited QTL regions were regarded as candidate genes for the multifoliolate phenotype. At the same time, using comparative expression analyses, the DEGs that are part of the regulatory mechanism behind the multifoliolate occurrence were identified to be stress-responsive genes. This study helped gain insights into the possible interplay between the genetic and environmental factors determining the multifoliolate phenotype in soybean.

## Materials and methods

### Plant materials

Wild soybean (*Glycine soja*) ‘W05’ was originally from Henan Province, China. Cultivated soybean (*Glycine max*) ‘C08’ was imported from the USA, with a cultivar name ‘Union [PI548622].’ The interspecific RIL population was derived from crossing W05 and C08 (Qi et al. 2014). After the F_2_ generation, single-seed descendants were propagated, and the seeds after F_7:8_ were used for sequencing and phenotyping. The reference genome of W05 and the whole-genome resequencing data of C08 were previously reported (Xie et al. 2019; Wang et al. 2021). The seeds used in this study were collected in 2020 at Xinxiang, Henan Province, China (35°080 N, 113°460 E).

### Phenotype investigation

We first observed the multifoliolate phenotype in the parental lines in an experimental field at The Chinese University of Hong Kong during the previous research. For phenotype investigation, the parental lines and RILs were planted in a greenhouse at The Chinese University of Hong Kong. Seeds were sown in plastic cups filled with vermiculite. Three biological replicates were grown in March–April 2021, September–October 2021, and August–September 2023, respectively. Four hundred and seven RILs with five seedlings each were grown in the first biological replicate, 406 RILs with ten seedlings each were grown in the second biological replicate, and 406 RILs with 20 seedlings each were grown in the third biological replicate, along with both parental lines. To record the occurrence of multifoliolate leaves in each line, the number of seedlings with multifoliolate leaves was counted at the V1 stage (when the first compound leaf had fully expanded), with “0” indicating no multifoliolate seedling, “1” indicating one multifoliolate seedling, “2” indicating two multifoliolate seedlings, and so on. The number of multifoliolate seedlings in each line was then used for subsequent analyses. Pearson correlation coefficients were calculated and plotted using the R/corrplot package. The yield-related traits, including flowering time, growth period, and 100-seed weight, were recorded when the RILs were planted in Xinxiang, Henan Province, China (35°080 N, 113°460 E), in 2020. Broad-sense heritability (*H*^*2*^) was calculated using the R/lme4 package with the following formula:

$$H2= \frac{{V}_{\text{g}}}{{V}_{\text{g}}+\frac{{V}_{\text{ge}}}{L}+\frac{{V}_{\varepsilon }}{\text{RL}}}$$

where V_g_ is the variance due to genotype, V_ge_ is the variance due to the interaction between genotype and environment, V_ɛ_ is the variance due to residual error, R is the number of replications per environment, and L is the number of environments.

### QTL mapping

QTL mapping was performed using a modified version of the R/qtl package (Broman et al. 2003), according to a method previously described (Huang et al. 2016; Xu et al. 2017; Wang et al. 2022b). One thousand permutations were conducted to determine the threshold for claiming a significant QTL at P < 0.05. A 1.5-LOD (logarithm of the odds) support interval was applied to estimate the confidence interval of each QTL. The bin map used for this RIL population was obtained from a previous work, and 6384 bin markers were included based on the reference genome of Williams 82 (version 4, Wm82v4) (Valliyodan et al. 2019; Wang et al. 2021, 2022b).

Besides the three biological replicates, the best linear unbiased prediction (BLUP) values across the three biological replicates were also included to perform the combined QTL analyses for the soybean multifoliolate trait. The BLUP values were calculated by the ‘lmer’ function in the R package lme4.

### Selection of bulks with extreme traits for BSR-seq

Two extreme groups, the high-multifoliolate frequency bulk (MUL) and the low-multifoliolate frequency bulk (TRI), were selected from within the RIL population based on the frequency of occurrence of the multifoliolate phenotype within each line. The MUL group contained 30 RILs with the highest multifoliolate frequencies, and the TRI group contained 30 RILs with the lowest multifoliolate frequencies among all the RILs. Since the basal compound leaf is developed from the shoot apical meristem, we collected shoot apical buds at the V0 stage (when the true leaf has fully opened) and the first compound leaf at the V1 stage (when it has fully opened) from each individual plant in the two extreme groups. Therefore, a total of four bulks (V0-MUL, V0-TRI, V1-MUL, and V1-TRI), each with three biological replicates, were used for further RNA-seq analyses.

### RNA extraction, library construction, and sequencing

Total RNA was extracted using the TRIzol reagent (Invitrogen, Carlsbad, CA) following the manufacturer’s instruction. Library construction and sequencing were performed by Novogene Co., Ltd. (Beijing, China) on the Illumina NovaSeq 6000 sequencing platform in the strand-specific 2 × 150 bp paired-end mode. Sequencing reads were checked for quality and filtered using fastp (version 0.23.2) with the default settings of a 4-nt sliding window and an average base quality of Q20 (Chen et al. 2018). Reads were aligned against the reference genome of Williams 82 (version 4, Wm82v4; Valliyodan et al. 2019) using Hisat2 (version 2.2.1; Kim et al. 2015). Duplicated reads were marked and removed using the ‘MarkDuplicates’ function (version 3.0.0) in Picard tools (https://broadinstitute.github.io/picard/). The abundance of transcripts was estimated using StringTie (version 2.2.1; Pertea et al. 2015) with reference to the Wm82v4 annotation.

### DEG analysis using RNA-seq data

Differential expression analyses were performed using the R package ‘edgeR’ (version 3.42.4; (Chen et al. 2008)). Genes were considered differentially expressed with the following criteria: |log_2_(fold change)|≥ 1 and *P* < 0.05.

Gene ontology (GO) and Kyoto Encyclopedia of Genes and Genomes (KEGG) enrichment analyses were performed with TBtools-II (Chen et al. 2023a). The GO enrichment bar graphs and gene expression heatmaps were drawn using TBtools-II (Chen et al. 2023a). KEGG pathway enrichment bubble plots were made using the R/ggplot2 package.

### SNP calling using BSR-seq data

The raw reads were filtered by Trimmomatic (version 0.36) according to previously published protocols (Bolger et al. 2014; Qu et al. 2023). All high-quality clean reads from each bulked group were mapped to the soybean Williams 82 reference genome (Wm82v4) using the BWA software (v0.7.12) with these parameters: ‘mem -t 4 -k 32 -M’ (Li 2013; Valliyodan et al. 2019). SAMtools (v1.3.1) was used for removing the PCR duplicates and sorting the alignment results (Li 2011). SNP and insertion/deletion (InDel) calling were performed using bcftools (v1.17; Danecek et al. 2021). Gene-based SNP and InDel identifications were carried out according to the Williams 82 genome annotation (Wm82.a4.v1) using the package ANNOVAR (v2013-06- 21; Wang et al. 2010). Upstream and downstream regions were defined as a 1-kb region upstream from the transcription start site (TSS) and a 1-kb region downstream from the transcription termination site (TTS), respectively. The SNP density graphs were drawn using RectChr (v1.37; https://github.co m/BGI-shenzhen/RectChr).

### Calculation of the SNP and ${\varvec{\Delta}}$ SNP indices

Before calculating the SNP and ΔSNP indices, the SNPs were filtered out if the sequencing depth of each bulk at each site was less than 3. The SNP index at each SNP position was calculated for both bulks according to the QTL-seq method (Takagi et al. 2013) and was calculated for all SNP positions. Any SNP position with an SNP index of < 0.3 in both bulks would be excluded from further analyses. In this study, the ΔSNP index was obtained by subtracting the SNP index of the MUL pool from the SNP index of the TRI pool for the multifoliolate trait. A 1-Mbp sliding window with 10-kbp increments was employed. Windows with fewer than 10 SNPs were skipped, and 100,000 iterations of the permutation test were performed to determine the 99% cutoff values.

## Results

### Phenotyping of the RIL population

**Fig. 1:**
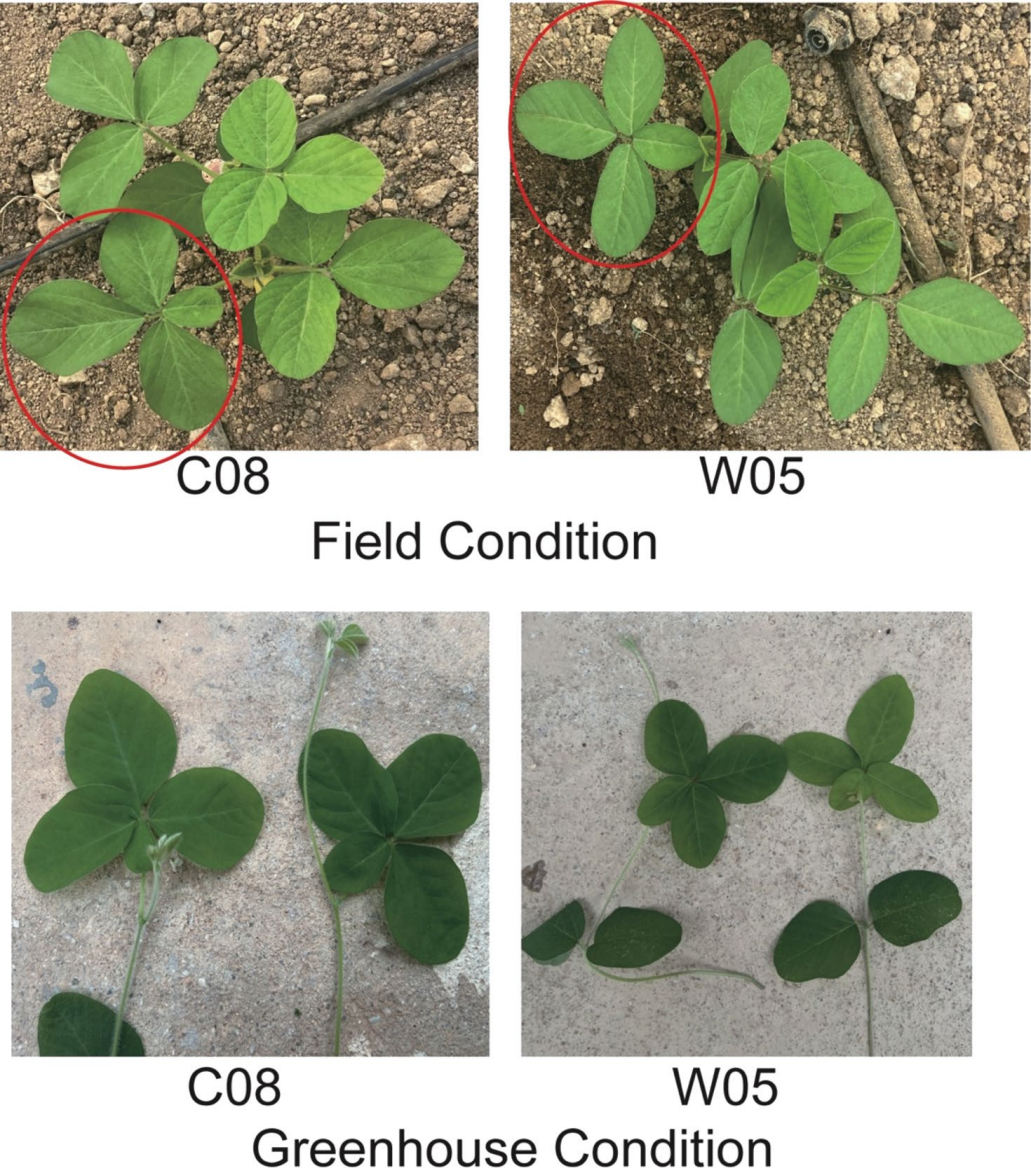
Multifoliolate phenotype in cultivated (C08) and wild (W05) soybean accessions grown in both field and greenhouse conditions

**Fig. 2:**
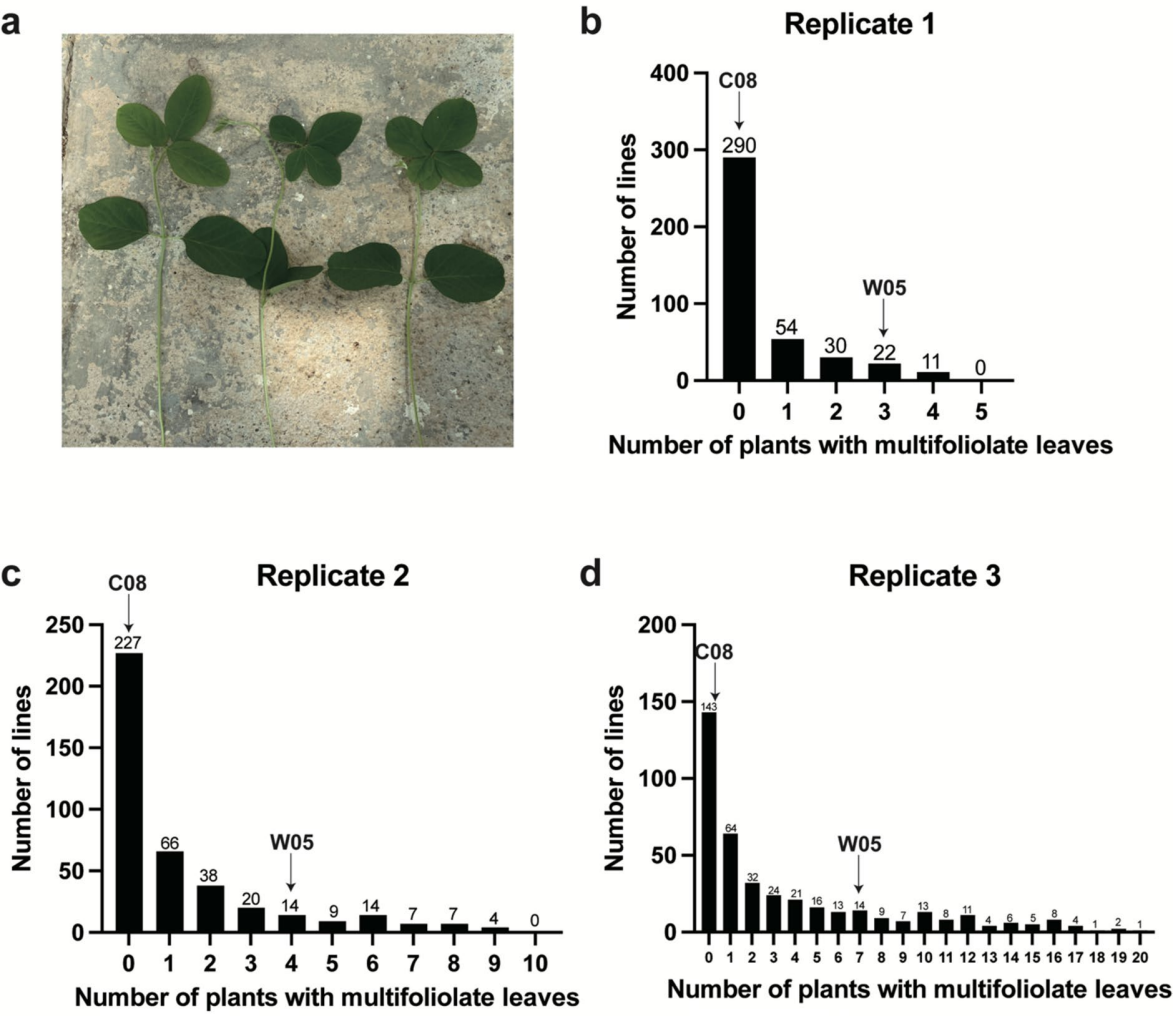
Distributions of the number of multifoliolate seedlings within the recombinant inbred line (RIL) population of C08 x W05. (a) A representative photo of trifoliolate, quadrifoliolate, and quinquefoliolate seedlings. (b-d) The distribution of the number of multifoliolate seedlings in each RIL in (b) replicate 1, (c) replicate 2, and (d) replicate 3. Black arrows indicate the values corresponding to those of C08 and W05

Under both greenhouse and field conditions, the multifoliolate phenotype was observed among the individual plants of both cultivated soybean C08 and wild soybean W05. The multifoliolate trait typically occurs at the basal compound leaf and is sporadic rather than fixed. In general, most of the multifoliolate leaves contained either four or five leaflets (Fig. 1). To identify the genetic basis underlying this phenomenon, an RIL population from the cross between C08 and W05 was phenotyped over three growing periods (as three biological replicates) under greenhouse conditions. Between the parental lines, there were more seedlings with multifoliolate leaves in W05 than in C08 in every replicate (Table S1** and **Fig. 2). As expected, seedlings of the RILs displayed trifoliolate, quadrifoliolate, and quinquefoliolate phenotypes (Fig. 2a). The number of individuals in each RIL having multifoliolate leaves was different across the population (Fig. 2b–d). The phenotypic data from the three biological replicates were significantly correlated (Fig. S1). The coefficient of variation (CV) of the occurrence of the multifoliolate phenotype was greater than 50%, suggesting that the phenotype has a high variability among different lines (Table S1). The broad-sense heritability (*H*^*2*^) was 0.6817, meaning that the multifoliolate phenotype was mostly determined by large-effect genetic factors (Table S1).

Furthermore, to explore the relationship between the multifoliolate phenotype and yield, we analyzed the correlations between it and various yield-related traits, including flowering time, growth period, and 100-seed weight. The results showed that the number of multifoliolate seedlings was significantly positively correlated with flowering time and growth period but not with the 100-seed weight (Fig. S2).

### Linkage mapping of the multifoliolate phenotype in soybean

**Fig. 3:**
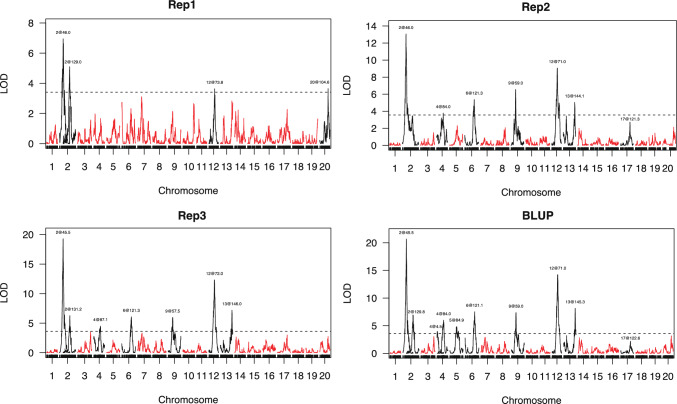
Quantitative trait locus (QTL) mapping results for the occurrence of multifoliolate seedlings in the recombinant inbred line (RIL) population of C08 x W05 for three biological replicates and in the best linear unbiased prediction (BLUP). The logarithm of odds (LOD) cutoff value was set by performing 1000 permutation tests. QTLs with LOD scores higher than the cutoff were regarded as putative QTLs. The chromosome number and genetic positions (in cM) of each QTL peak are shown. Rep1/2/3, replicates 1/2/3

The phenotypic data for three biological replicates and the BLUP of the three biological replicates were used in QTL mapping. A total of ten QTLs were identified as significantly associated with the multifoliolate phenotype in soybean, with four to nine QTLs detected in each biological replicate and the BLUP (Fig. 3** and **Table S2), distributed on eight chromosomes. Among them, *qMF-2–1* was detected in all three replicates and BLUP, explaining 6.65–13.94% of the phenotypic variation, with LOD values between 6.95 and 20.68 and an additive effect ranging from -0.265 to -1.817. *qMF-12* was also detected in all three replicates and BLUP, explaining 3.41–8.55% of the phenotypic variation, with LOD values between 3.64 and 14.25 and an additive effect of 0.19–1.421. Since *qMF-2–1* and *qMF-12* were detected in all replicates, we considered these two loci to be stable contributors to the multifoliolate trait. For others, *qMF-2–2*, *qMF-4–2*, *qMF-6*, *qMF-9*, and *qMF-13* were detected in two replicates and the BLUP, while *qMF-4–1*, *qMF-5*, and *qMF-20* were only detected in one replicate or the BLUP (Fig. 3** and **Table S2). The QTLs found in each of the four sets of data (three biological replicates and BLUP) collectively explained approximately 18.32%, 35.24%, 42.61%, and 48.31% of all phenotypic variation, respectively (Table S2). The analysis of additive effects revealed that the positive genetic effects were contributed by the wild parent W05 (Table S2).

The confidence regions of the ten QTLs detected in linkage mapping were decided by the common regions detected in all replicates (Table 1). The minimum range was 0.3 Mb in *qMF-2–1*, while the maximum range was 34.6 Mb in *qMF-4–2* (Table 1). Based on the gene annotations of the Williams 82 reference genome (Wm82v4) from Wildsoydb DataHub (https://datahub.wildsoydb.org/) (Xiao et al. 2022), 2420 genes in total were identified to reside in the ten QTLs (Table 1).

**Table 1:** The delineated regions of quantitative trait loci (QTLs) detected by linkage mapping

QTL name	Biological replicates	Chromosome	Start position	End position	Range (Mb)	Number of genes
*qMF-2–1*	Rep1, Rep2, Rep3, and BLUP	Gm02	5,550,000	5,850,000	0.3	44
*qMF-2–2*	Rep1, Rep3, and BLUP	Gm02	33,000,000	40,350,000	7.35	154
*qMF-4–1*	BLUP	Gm04	0	2,650,000	2.65	336
*qMF-4–2*	Rep2, Rep3, and BLUP	Gm04	9,650,000	44,250,000	34.6	641
*qMF-5*	BLUP	Gm05	29,050,000	36,100,000	7.05	601
*qMF-6*	Rep2, Rep3, and BLUP	Gm06	17,300,000	20,700,000	3.4	138
*qMF-9*	Rep2, Rep3, and BLUP	Gm09	5,650,000	6,450,000	0.8	51
*qMF-12*	Rep1, Rep2, Rep3, and BLUP	Gm12	12,300,000	20,050,000	7.75	206
*qMF-13*	Rep2, Rep3, and BLUP	Gm13	42,800,000	43,350,000	0.55	70
*qMF-20*	Rep1	Gm20	43,200,000	44,750,000	1.55	179

BLUP, best linear unbiased prediction and Rep1/2/3, biological replicate 1/2/3.

### Identification of SNPs associated with the multifoliolate phenotype by BSR-seq

To identify the SNPs associated with the multifoliolate phenotype, BSR-seq was utilized, assuming that the phenotype is caused by sequence variations in the expressed genes. Based on the frequencies of multifoliolate occurrence in the RILs, 30 lines with the highest multifoliolate occurrence (MUL) and 30 lines with the lowest occurrence (TRI), as defined by the number of individual plants having multifoliolate leaves within each RIL, were selected for BSR-seq. Four RNA bulks (V0-MUL, V0-TRI, V1-MUL, and V1-TRI), with three biological replicates each, were constructed from shoot apical buds at V0 (true leaf stage) and fully opened compound leaves at V1 (first compound leaf stage), and were sequenced independently on the Illumina NovaSeq 6000 platform. Approximately 44.6 million–70.6 million clean reads were obtained from each sample pool (Table S3).

**Fig. 4:**
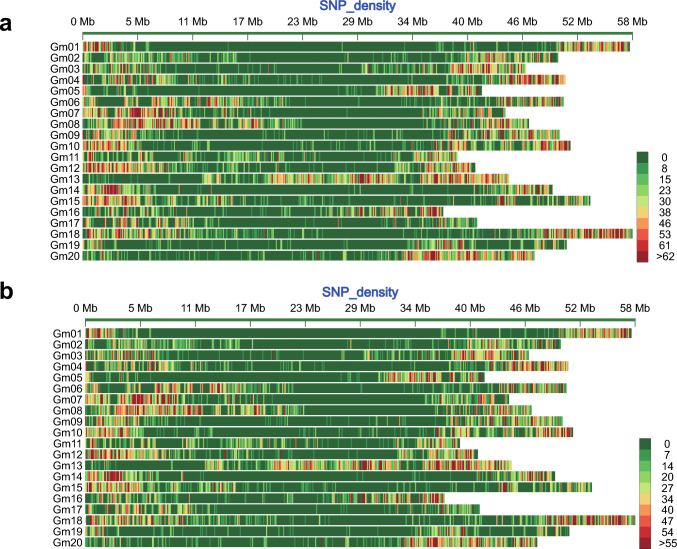
The single-nucleotide polymorphism (SNP) density between the high-multifoliolate frequency bulk (MUL) and the low-multifoliolate frequency bulk (TRI) on the soybean chromosomes. (a) Distribution of the SNPs between V0-MUL and V0-TRI. (b) Distribution of the SNPs between V1-MUL and V1-TRI. The color of each bar represents the number of SNPs within a 100-kbp window based on the color key. The horizontal axis represents the length of the chromosomes. V0, shoot apical bud from the true leaf stage and V1, leaf tissue from the first compound leaf

After filtering, mapping, SNP calling, and a second filtering, 365,589 SNP positions were identified among the four RNA bulks. In order to pinpoint the genetic regions corresponding to the multifoliolate phenotype within the whole genome, the SNPs between MUL and TRI were examined. In total, 144,262 SNPs were identified between the V0-MUL and V0-TRI bulks, and 122,707 SNPs were identified between the V1-MUL and V1-TRI bulks (Fig. 4).

Based on the identified SNPs, we calculated the statistical confidence intervals of the ΔSNP indices for all the SNP positions (*P* < 0.01) (Fig. S3). A comparative analysis between V0-MUL and V0-TRI revealed 23 significant association loci on chromosomes 2, 6, 8, 9, 12, 14, 17, 19, and 20, harboring 621 genes (Table S4). A similar comparative analysis between V1-MUL and V1-TRI yielded 13 significant loci mapped to chromosomes 2, 4, 8, 12, and 20, containing 148 genes (Table S5).

### Screening for candidate causal genes controlling the multifoliolate phenotype by integrating the results from both linkage mapping and BSR-seq

To increase the confidence of candidate gene identification, only the multifoliolate loci identified by both linkage mapping and BSR-seq were selected. Three highly overlapped regions were thus found in the V0 sample pools, including *qMF-2–2* and *qMF-6*, containing 112 genes altogether (Table 2). Among these genes, only 49 genes were expressed in both V0 BSR-seq bulks (FPKM > 1) (Table S6). Functional annotations of these genes revealed that some of them were related to leaf development. For instance, *Glyma.06G204300* encodes a transcription factor TCP5, which controls leaf margin development by regulating *KNOTTED-LIKE HOMEOBOX* (*KNOX*), a group of genes mainly expressed in the leaf primordium responsible for leaf morphology (Yu et al. 2021). Another gene common to both V0 bulks, *Glyma.06G204400*, encodes LONGIFOLIA 2 (LNG2), which is involved in regulating leaf morphology by promoting cell expansion in *Arabidopsis* (Lee et al. 2006). In addition, by comparing between the published genomes of W05 and Williams 82 (which shared the same recurrent parent with C08 used in this study), these two genes showed nonsynonymous polymorphisms within the coding region between the two parental lines (Table S6). The analyses of the BSR-seq data with respect to the relative SNP frequencies between the two bulks showed that the higher alternative genotype frequencies in V0-MUL were attributable to W05, while the higher reference genotype frequencies in V0-TRL were attributable to C08 (Table S7).

**Table 2:** Candidate quantitative trait loci (QTLs) from the joint analyses of linkage mapping and bulked segregant RNA sequencing (BSR-seq)

Chromosome	QTL name	Detection source	Start position	End position	Number of overlapping genes
Gm02	*qMF-2–2*	Linkage mapping	33,000,000	40,350,000	
		BSR-seq (V0)	32,820,000	33,960,000	18
		BSR-seq (V0)	34,440,000	35,800,000	12
		BSR-seq (V1)	34,610,000	35,900,000	12
Gm04	*qMF-4–2*	Linkage mapping	9,650,000	44,250,000	
		BSR-seq (V1)	28,670,000	30,180,000	13
		BSR-seq (V1)	33,460,000	34,500,000	7
		BSR-seq (V1)	35,890,000	36,950,000	5
Gm06	*qMF-6*	Linkage mapping	17,300,000	20,700,000	
		BSR-seq (V0)	17,560,000	19,470,000	82
Gm12	*qMF-12*	Linkage mapping	12,300,000	20,050,000	
		BSR-seq (V1)	11,650,000	12,720,000	9

V0, shoot apical bud from the true leaf stage and V1, leaf tissue from the first compound leaf.

In the V1 sample bulks, there were five regions identified by both detection methods, including *qMF-2–2*, *qMF-4–2*, and *qMF-12*, containing 46 genes in total, but only 18 of the genes were expressed in the V1 sample bulks (Table 2 and Table S6), and none of them were related to leaf development.

### Transcriptomic analyses of the multifoliolate phenotype in soybean

**Fig. 5:**
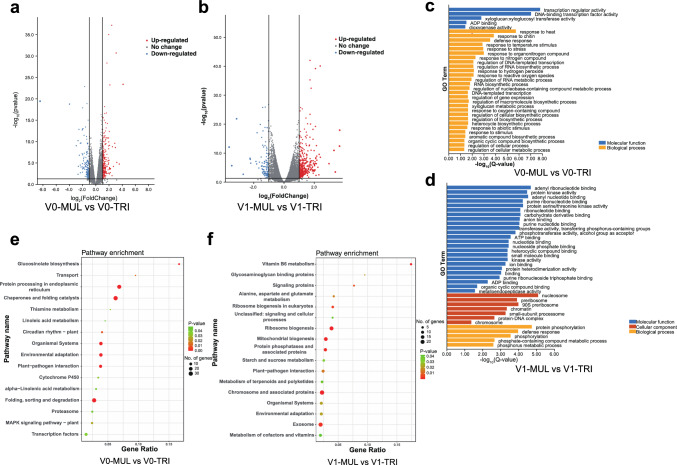
Differential expression analyses of RNA-seq data. (a-b) Volcano plots showing the number of differentially expressed genes (DEGs) based on log_2_FoldChange(MUL/TRI) and P value in (a) V0-MUL versus V0-TRI and (b) V1-MUL versus V1-TRI. Red dots indicate the up-regulated genes, and blue dots represent the down-regulated genes. (c-d) Gene ontology (GO) analyses of the DEGs in (c) V0-MUL versus V0-TRI and (d) V1-MUL versus V1-TRI, with false discovery rate (FDR) < 0.05. (e–f) Kyoto Encyclopedia of Genes and Genomes (KEGG) pathway enrichment analyses of the DEGs in (e) V0-MUL versus V0-TRI and (f) V1-MUL versus V1-TRI, with P value < 0.05. MUL, high-multifoliolate frequency bulk; TRI, low-multifoliolate frequency bulk; V0, shoot apical bud from the true leaf stage; and V1, leaf tissue from the first compound leaf

In examining differential gene expressions between the TRI and MUL bulks at the same developmental stage, 436 DEGs were detected between V0-MUL and V0-TRI, among which 176 genes were down-regulated and 260 were up-regulated in MUL. Meanwhile, 551 DEGs were found between V1-MUL and V1-TRI, where 113 genes were down-regulated and 438 were up-regulated in MUL (Fig. 5a, b). To better understand the gene regulation of the multifoliolate phenotype, all DEGs were annotated using the GO and KEGG databases (Fig. 5c–f). The DEGs between V0-MUL and V0-TRI were significantly enriched in these GO terms: ‘response to heat,’ ‘response to chitin,’ and ‘defense response’ (Fig. 5c). Between V1-MUL and V1-TRI, the DEGs were largely enriched in the GO terms ‘protein phosphorylation’ and ‘defense response’ (Fig. 5d). The KEGG pathways, ‘protein processing,’ ‘environmental adaptation,’ ‘plant-pathogen interaction,’ and ‘MAPK signaling pathway-plant,’ were significantly enriched among the DEGs between V0-MUL and V0-TRI, while ‘ribosome biogenesis,’ ‘protein phosphatases and associated proteins,’ ‘plant-pathogen interaction,’ and ‘starch and sucrose metabolism’ were pathways enriched among the DEGs between V1-MUL and V1-TRI (Fig. 5e, f).

**Fig. 6:**
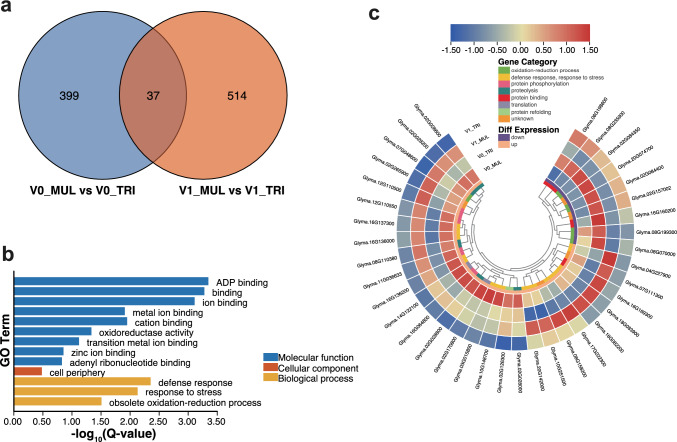
The overlapping differentially expressed genes (DEGs) between V0-MUL vs V0-TRI and V1-MUL vs V1-TRI. (a) A Venn diagram of the number of DEGs identified in the shoot apical bud analyses (V0_MUL vs V0_TRI) and in the leaf tissue analyses (V1_MUL vs V1_TRI). (b) Gene ontology (GO) terms of 37 overlapping DEGs (with false discovery rate [FDR] < 0.05). (c) A gene expression heatmap of 37 overlapping DEGs in all four sample pools. The DEGs were sorted as either down-regulated or up-regulated in the MUL sample pool, as well as according to eight gene categories. MUL, high-multifoliolate frequency bulk; TRI, low-multifoliolate frequency bulk; V0, shoot apical bud from the true leaf stage; and V1, leaf tissue from the first compound leaf

When comparing the DEGs between the V0 and V1 pools, 37 DEGs were common to the V0-MUL/V0-TRI and the V1-MUL/V1-TRI comparisons, constituting the group of genes likely to be involved in regulating the multifoliolate phenotype at both developmental stages (Fig. 6a). These DEGs were significantly enriched in the GO terms ‘defense response,’ ‘response to stress,’ and ‘obsolete oxidation–reduction process’ (Fig. 6b). Among these DEGs, nine were down-regulated while the others were up-regulated in MUL at both stages (Fig. 6c). These 37 genes were enriched in eight gene categories, with 11 up-regulated genes enriched in ‘defense response’ and ‘response to stress,’ suggesting that the occurrence of the multifoliolate phenotype may be highly linked to stress responses.

## Discussion

The soybean leaf is typically trifoliolate, but multifoliolate such as quadri- and quinque-foliolate leaves has also been reported in spontaneous and artificial mutants. In this study, we observed that sporadic multifoliolate leaves can be found in both wild and cultivated soybeans, as well as in the RIL population from a cross between a wild parent and a cultivar. High correlation coefficients among the three biological replicates and the high broad-sense heritability indicate that this phenomenon is largely controlled by genetic factors. However, the genetic mechanism behind the multifoliolate phenotype has been poorly understood.

From our results, the frequencies of multifoliolate seedlings in W05 were higher than those in C08 in every replicate (Fig. 2). This was consistent with the positive genetic contribution from W05 based on the additive effect calculations (Table S2). In addition, based on the BSR-seq results, the higher frequencies of the alternative genotype in MUL were attributable to W05, while the higher frequencies of the reference genotype in TRI were attributable to C08 (Table S7). All these results together indicated that the multifoliolate phenotype was inherited from wild soybeans, which was also described by a previous study (Wang et al. 2001).

The development of leaves is a complicated process regulated by both environmental and genetic factors. Earlier studies have found that the multifoliolate phenomenon in soybean leaves was controlled by the loci *Lf1*, *Lf2*, *Lf3*, *Lf4*, and *Lf5* (Fehr 1972; Wang et al. 2001). So far, *Lf1* and *Lf2* have been identified on chromosomes 8 and 11, respectively (Muehlbauer et al. 1991; Seversike et al. 2008; Jeong et al. 2017). Later, using three RIL populations, Orf et al. (2006) delineated the QTLs related to the multifoliolate phenotype, including the frequency of occurrence in individual plants and the number of nodes expressing the phenotype, in 17 regions of the soybean genome. Here, we performed QTL mapping on a biparental RIL population and identified ten multifoliolate-related loci on chromosomes 2, 4, 5, 6, 9, 12, 13, and 20 (Fig. 3**)**, different from the findings of the previous studies. This result suggests that the QTLs obtained in all these studies, including ours, are dependent on the specific genetic populations and environmental factors. Also, here we focused on the multifoliolate development at the first node, which is different from the other studies.

Notably, the multifoliolate phenotype was a quantitative trait, with different lines having different multifoliolate frequencies (Fig. 2). From our results, the number of QTLs detected in Rep1 was fewer than in Rep2 and 3, and *qMF-20* could be only found in Rep1 but not in Rep2 or 3 (Fig. 3). This result may be caused by the limitation due to the lower number of individuals in Rep1. Nevertheless, the QTLs detected in Rep2 and 3 were almost the same, indicating that using 10 individuals per line in Rep2 for phenotypic investigation was enough to reflect the frequencies of multifoliolate plants for genetic mapping in this RIL population.

Furthermore, as discovered in this study, the multifoliolate phenotype is governed by multiple QTLs, with many candidate genes residing within these regions. Therefore, it can be difficult to pinpoint the precise causal genes. It has been reported that integrating different QTL analysis approaches could improve the efficiency of genetic dissection by increasing the confidence of QTL detection (Chen et al. 2023b), such as the studies on soybean (Li et al. 2020; Song et al. 2023). Hence, in addition to linkage mapping, we also performed BSR-seq based on high-multifoliolate frequency and low-frequency RILs. Twenty-three genetic regions were thus identified from the BSR-seq analyses between V0-MUL and V0-TRI, and 13 regions were obtained from the analyses between V1-MUL and V1-TRI, respectively. Among them, some regions were common to both linkage mapping and BSR-seq results, providing us with a high degree of confidence for having isolated the regions responsible for the multifoliolate phenotype.

In other crops, it has been demonstrated that the formation of compound leaves is determined by complex genetic networks (Hao et al. 2022), including the *KNOX* genes that play significant roles in determining leaf complexity (Bharathan et al. 2002; Ma and Li 2022; Wang et al. 2022a). In *Medicago truncatula*, it was reported that a C2H2 zinc-finger transcription factor, PALM1, determined the development of the typical trifoliolate leaves, and *palm1* mutants had the quinquefoliolate phenotype (Chen et al. 2010). Recently, through BSA, a causal gene related to the multi-pinnate leaf phenotype in chickpea was identified as an ortholog of *PALM1* (Liu et al. 2023). However, in our study, none of the *KNOX* or *PALM1* genes were discovered in the multifoliolate QTLs, and they were not differentially expressed between the high- and low-multifoliolate occurrence bulks in either the shoot apical bud or leaf tissue. Therefore, there might be other genes involved in regulating the multifoliolate phenotype in our genetic population. In *Arabidopsis*, the CIN-clade TCP gene, *TCP5*, was reported to control leaf margin development by regulating the KNOX and BEL-like transcription factors (Yu et al. 2021). *LONGIFOLIA1* (*LNG1*) and *LONGIFOLIA2* (*LNG2*) were shown to determine leaf morphology by promoting longitudinal cell elongation in *Arabidopsis* (Lee et al. 2006). According to our results, *Glyma.06G204300* (encoding TCP5) and *Glyma.06G204400* (encoding LNG2) resided in *qMF-6* and were detected by both linkage mapping and BSR-seq in the V0 sample pools, so these genes may be important in regulating the occurrence of multifoliolate leaves in soybean. It is important to note that these two genes exhibited nonsynonymous polymorphisms between parental lines, but their expression levels did not show significant differences between the two bulks (Table S6). This suggested that the multifoliolate phenotype might be related to these polymorphisms which could have potentially contributed to alterations in protein functions but not to changes in gene expression levels.

The soybean leaf is genetically programmed to be typically trifoliolate. Nonetheless, environmental stimuli could bring about the multifoliolate morphology in some instances. A previous study suggested that the multifoliolate trait is an indicator of the plant’s response to its environment since some plants frequently develop multifoliolate leaves at certain nodes, under specific environmental conditions (Orf et al. 2006). Similarly, another study showed that the expression of the multifoliolate phenotype in *Trifolium alexandrinum* L. is dependent on the growth stage and environmental conditions (Malaviya et al. 2021). However, these studies did not investigate the underlying molecular mechanism for the phenomenon. Here, based on transcriptomics, we found a connection between the multifoliolate phenotype and stress, as demonstrated in the enriched GO terms such as ‘response to heat,’ ‘defense response,’ ‘response to stress,’ and ‘environmental adaptation’ among the DEGs in the high-multifoliolate frequency RILs in both shoot apical buds (V0) and leaf tissues (V1). This suggested that the occurrence of the multifoliolate phenotype could be a result of the interactions between genes and the environment.

It would be a significant discovery if the understanding on leaf development can lead to improved crop yield. Compared to the trifoliolate phenotype, the multifoliolate leaf possesses a larger surface area, which is likely to receive more sunlight, leading to a higher photosynthetic yield. The previous study on mung bean showed that yield and seed weight varied depending on different leaflet morphologies (Soehendi et al. 2007). In our results, no significant correlation was identified between the multifoliolate phenotype and 100-seed weight (Fig. S2), which may be due to the fact that the multifoliolate phenotype was only found in the first compound leaf, which contributes to just a small portion of the total leaf area. Interestingly though, the multifoliolate phenotype showed significant correlations with flowering time and growth period (Fig. S2) which are also important components contributing to the final crop yield. This indicated that the multifoliolate phenotype is indirectly related to yield by affecting flowering time and growth period. Thus, it is worth further investigations into the pleiotropic effects of the multifoliolate trait-related loci on flowering time and soybean yield in the future. Moreover, considering the genetic contribution of the multifoliolate phenotype from wild soybeans, the utilization of genetic resources from wild germplasms to improve the yield of cultivated soybeans should be a promising approach in soybean breeding.

In this study, we combined linkage mapping and BSR-seq to explore the genetic regulation of the multifoliolate phenotype in soybean. Future research on how the causal genes identified here respond to environmental conditions could further improve our understanding of the molecular mechanism behind the multifoliolate phenotype in soybean.

## Supplementary Information

Below is the link to the electronic supplementary material.

Supplementary file1 (DOCX 15 KB)

Supplementary file2 (XLSX 13 KB)

Supplementary file3 (XLSX 11 KB)

Supplementary file4 (DOCX 18 KB)

Supplementary file5 (DOCX 17 KB)

Supplementary file6 (XLSX 17 KB)

Supplementary file7 (XLSX 28 KB)

Supplementary file8 (DOCX 6836 KB)

## Data Availability

All data generated or analyzed in this study are included in this published article and its supplementary information files. The raw sequences generated in this study were deposited at the NCBI under accession number PRJNA1043832.

## References

[CR1] Bharathan G, Goliber TE, Moore C et al (2002) Homologies in leaf form inferred from *KNOXI* gene expression during development. Science 296:1858–1860. 10.1126/science.1070343 12052958 10.1126/science.1070343

[CR2] Bolger AM, Lohse M, Usadel B (2014) Trimmomatic: A flexible trimmer for Illumina sequence data. Bioinformatics 30:2114–2120. 10.1093/bioinformatics/btu170 24695404 10.1093/bioinformatics/btu170 PMC4103590

[CR3] Broman KW, Wu H, Sen Ś, Churchill GA (2003) R/qtl: QTL mapping in experimental crosses. Bioinformatics 19:889–890. 10.1093/bioinformatics/btg112 12724300 10.1093/bioinformatics/btg112

[CR4] Chen J, Yu J, Ge L et al (2010) Control of dissected leaf morphology by a Cys(2)His(2) zinc finger transcription factor in the model legume *Medicago truncatula*. Proc Natl Acad Sci U S A 107:10754–10759. 10.1073/pnas.1003954107 20498057 10.1073/pnas.1003954107 PMC2890821

[CR5] Chen S, Zhou Y, Chen Y, Gu J (2018) Fastp: An ultra-fast all-in-one FASTQ preprocessor. Bioinformatics 34:i884–i890. 10.1093/bioinformatics/bty560 30423086 10.1093/bioinformatics/bty560 PMC6129281

[CR6] Chen C, Wu Y, Li J et al (2023a) TBtools-II: A “one for all, all for one” bioinformatics platform for biological big-data mining. Mol Plant 16:1733–1742. 10.1016/j.molp.2023.09.010 37740491 10.1016/j.molp.2023.09.010

[CR7] Chen Y, Xiong Y, Hong H et al (2023b) Genetic dissection of and genomic selection for seed weight, pod length, and pod width in soybean. Crop J 11:832–841. 10.1016/j.cj.2022.11.006

[CR8] Chen Y, McCarthy D, Baldoni P, et al (2008) edgeR: differential analysis of sequence read count data User’s Guide. 1–138

[CR9] Danecek P, Bonfield JK, Liddle J et al (2021) Twelve years of SAMtools and BCFtools. Gigascience 10:1–4. 10.1093/gigascience/giab008 10.1093/gigascience/giab008 PMC7931819 33590861

[CR10] Du H, Zhu J, Su H et al (2017) Bulked segregant RNA-seq reveals differential expression and SNPs of candidate genes associated with waterlogging tolerance in maize. Front Plant Sci 8:1–13. 10.3389/fpls.2017.01022 28659961 10.3389/fpls.2017.01022 PMC5470080

[CR11] Fehr WR (1972) Genetic Control of Leaflet Number in Soybeans. Crop Sci 12:221–224. 10.2135/cropsci1972.0011183x001200020023x

[CR12] Gao Y, Du L, Ma Q et al (2022) Conjunctive Analyses of Bulk Segregant Analysis Sequencing and Bulk Segregant RNA Sequencing to Identify Candidate Genes Controlling Spikelet Sterility of Foxtail Millet. Front Plant Sci 13:1–14. 10.3389/fpls.2022.842336 10.3389/fpls.2022.842336 PMC9047506 35498640

[CR13] Hao N, Cao J, Wang C et al (2022) Understanding the molecular mechanism of leaf morphogenesis in vegetable crops conduces to breeding process. Front Plant Sci 13:1–10. 10.3389/fpls.2022.971453 10.3389/fpls.2022.971453 PMC9773389 36570936

[CR14] Huang C, Chen Q, Xu G et al (2016) Identification and fine mapping of quantitative trait loci for the number of vascular bundle in maize stem. J Integr Plant Biol 58:81–90. 10.1111/jipb.12358 25845500 10.1111/jipb.12358 PMC5034846

[CR15] Huang CC, Lin CH, Lin YC, Chang HX (2024) Application of bulk segregant RNA-Seq (BSR-Seq) and allele-specific primers to study soybean powdery mildew resistance. BMC Plant Biol 24:155. 10.1186/s12870-024-04822-1 38424508 10.1186/s12870-024-04822-1 PMC10905810

[CR16] Jeong SC, Kim JH, Bae DN (2017) Genetic analysis of the *Lf1* gene that controls leaflet number in soybean. Theor Appl Genet 130:1685–1692. 10.1007/s00122-017-2918-0 28516383 10.1007/s00122-017-2918-0

[CR17] Juan NA, Sheaffer CC, Barnes DK et al (1993a) Leaf and stem traits and herbage quality of multifoliolate alfalfa. Agron J 85:1121–1127. 10.2134/agronj1993.00021962008500060005x

[CR18] Juan NA, Sheaffer CC, Barnes DK (1993b) Temperature and Photoperiod Effects on Multifoliolate Expression and Morphology of Alfalfa. Crop Sci 33:573–578. 10.2135/cropsci1993.0011183x003300030030x

[CR19] Kim D, Langmead B, Salzberg SL (2015) HISAT: a fast spliced aligner with low memory requirements. Nat Methods 12:357–360. 10.1038/nmeth.3317 25751142 10.1038/nmeth.3317 PMC4655817

[CR20] Lee YK, Kim GT, Kim IJ et al (2006) *LONGIFOLIA1* and *LONGIFOLIA2*, two homologous genes, regulate longitudinal cell elongation in *Arabidopsis*. Development 133:4305–4314. 10.1242/dev.02604 17038516 10.1242/dev.02604

[CR21] Li H (2011) A statistical framework for SNP calling, mutation discovery, association mapping and population genetical parameter estimation from sequencing data. Bioinformatics 27:2987–2993. 10.1093/bioinformatics/btr509 21903627 10.1093/bioinformatics/btr509 PMC3198575

[CR22] Li R, Jiang H, Zhang Z et al (2020) Combined linkage mapping and bsa to identify qtl and candidate genes for plant height and the number of nodes on the main stem in soybean. Int J Mol Sci. 10.3390/ijms21010042 31861685 10.3390/ijms21010042 PMC6981803

[CR23] Li H (2013) Aligning sequence reads, clone sequences and assembly contigs with BWA-MEM. 00: 1-3

[CR24] Liu Y, Yang Y, Wang R et al (2023) Control of compound leaf patterning by *MULTI-PINNATE LEAF1* (*MPL1*) in chickpea. Nat Commun. 10.1038/s41467-023-43975-9 38062032 10.1038/s41467-023-43975-9 PMC10703836

[CR25] Ma J, Li H (2022) The formation of shapes: interplay of genes during leaf development processes. Forests 13:1–14. 10.3390/f13101726

[CR26] Majeed A, Johar P, Raina A et al (2022) Harnessing the potential of bulk segregant analysis sequencing and its related approaches in crop breeding. Front Genet. 10.3389/fgene.2022.944501 36003337 10.3389/fgene.2022.944501 PMC9393495

[CR27] Malaviya DR, Roy AK, Kaushal P et al (2021) Phenotype study of multifoliolate leaf formation in *trifolium alexandrinum* L. PeerJ. 10.7717/peerj.10874 33717683 10.7717/peerj.10874 PMC7936568

[CR28] Muehlbauer GJ, Staswick PE, Specht JE et al (1991) RFLP mapping using near-isogenic lines in the soybean [*Glycine max* (L.) Merr.]. Theor Appl Genet 81:189–198. 10.1007/BF00215722 24221202 10.1007/BF00215722

[CR29] Orf JH, Chase K, Specht J et al (2006) Abnormal leaf formation in soybean: genetic and environmental effects. Theor Appl Genet 113:137–146. 10.1007/s00122-006-0280-8 16783594 10.1007/s00122-006-0280-8

[CR30] Pertea M, Pertea GM, Antonescu CM et al (2015) stringtie enables improved reconstruction of a transcriptome from RNA-seq reads. Nat Biotechnol 33:290–295. 10.1038/nbt.3122 25690850 10.1038/nbt.3122 PMC4643835

[CR31] Qi X, Li MW, Xie M et al (2014) Identification of a novel salt tolerance gene in wild soybean by whole-genome sequencing. Nat Commun 5:1–11. 10.1038/ncomms5340 10.1038/ncomms5340 PMC4104456 25004933

[CR32] Qu C, Zhu M, Hu R et al (2023) Comparative genomic analyses reveal the genetic basis of the yellow-seed trait in *Brassica napus*. Nat Commun. 10.1038/s41467-023-40838-1 37626056 10.1038/s41467-023-40838-1 PMC10457299

[CR33] Ramirez-Gonzalez RH, Segovia V, Bird N et al (2015) RNA-Seq bulked segregant analysis enables the identification of high-resolution genetic markers for breeding in hexaploid wheat. Plant Biotechnol J 13:613–624. 10.1111/pbi.12281 25382230 10.1111/pbi.12281

[CR34] Seversike TM, Ray JD, Shultz JL, Purcell LC (2008) Soybean molecular linkage group B1 corresponds to classical linkage group 16 based on map location of the *lf2* gene. Theor Appl Genet 117:143–147. 10.1007/s00122-008-0759-6 18392801 10.1007/s00122-008-0759-6

[CR35] Soehendi R, Chanprame S, Toojinda T et al (2007) Genetics, agronomic, and molecular study of leaflet mutants in mungbean(*Vigna radiata* (L.) Wilczek). J Crop Sci Biotechnol 10:193–200

[CR36] Song J, Xu R, Guo Q et al (2023) An omics strategy increasingly improves the discovery of genetic loci and genes for seed-coat color formation in soybean. Mol Breed 43:1–16. 10.1007/s11032-023-01414-z 37663546 10.1007/s11032-023-01414-z PMC10471558

[CR37] Takagi H, Abe A, Yoshida K et al (2013) QTL-seq: Rapid mapping of quantitative trait loci in rice by whole genome resequencing of DNA from two bulked populations. Plant J 74:174–183. 10.1111/tpj.12105 23289725 10.1111/tpj.12105

[CR38] Valliyodan B, Cannon SB, Bayer PE et al (2019) Construction and comparison of three reference-quality genome assemblies for soybean. Plant J 100:1066–1082. 10.1111/tpj.14500 31433882 10.1111/tpj.14500

[CR39] Wang K, Li F, Zhou T, Xu Z (2001) Inheritance of a five leaflet character arising from wild soybean (*Glycine soja* Sieb. et Zucc. ) in soybeans (*G. max* (L.) Merr.). Soybean Sci 20:22–25

[CR40] Wang J, Wu Y, Yu Z (2007) A new soybean [*Glycine max* (L.) Merr.] mutant with multifoliolate compound leaf acquired by ion beam irradiation. Nucl Instruments Methods Phys Res Sect B Beam Interact with Mater Atoms 255:326–330. 10.1016/j.nimb.2006.12.180

[CR41] Wang K, Li M, Hakonarson H (2010) ANNOVAR: Functional annotation of genetic variants from high-throughput sequencing data. Nucleic Acids Res 38:1–7. 10.1093/nar/gkq603 20601685 10.1093/nar/gkq603 PMC2938201

[CR42] Wang X, Li MW, Wong FL et al (2021) Increased copy number of gibberellin 2-oxidase 8 genes reduced trailing growth and shoot length during soybean domestication. Plant J 107:1739–1755. 10.1111/tpj.15414 34245624 10.1111/tpj.15414

[CR43] Wang Y, Strauss S, Liu S et al (2022a) The cellular basis for synergy between *RCO* and *KNOX1* homeobox genes in leaf shape diversity. Curr Biol 32:3773-3784.e5. 10.1016/j.cub.2022.08.020 36029772 10.1016/j.cub.2022.08.020

[CR44] Wang Z, Huang C, Niu Y et al (2022b) QTL analyses of soybean root system architecture revealed genetic relationships with shoot-related traits. Theor Appl Genet 135:4507–4522. 10.1007/s00122-022-04235-4 36422673 10.1007/s00122-022-04235-4

[CR45] Xiao Z, Wang Q, Li M-W et al (2022) Wildsoydb DataHub : an online platform for accessing soybean multiomic datasets across multiple reference genomes. Plant Physiol 00:1–4 10.1093/plphys/kiac419 PMC9706425 36063461

[CR46] Xie M, Chung CYL, Li MW et al (2019) A reference-grade wild soybean genome. Nat Commun 10:1–12. 10.1038/s41467-019-09142-9 30872580 10.1038/s41467-019-09142-9 PMC6418295

[CR47] Xu G, Wang X, Huang C et al (2017) Complex genetic architecture underlies maize tassel domestication. New Phytol 214:852–864. 10.1111/nph.14400 28067953 10.1111/nph.14400 PMC5363343

[CR48] Yancheva C, Petkova D, Sevov A (2012) Studies on Quality of Multifoliolate Alfalfa. Sci Pap Ser A Agron 261–264

[CR49] Yu H, Zhang L, Wang W et al (2021) TCP5 controls leaf margin development by regulating KNOX and BEL-like transcription factors in Arabidopsis. J Exp Bot 72:1809–1821. 10.1093/jxb/eraa569 33258902 10.1093/jxb/eraa569

[CR50] Zhan H, Wang Y, Zhang D et al (2021) RNA-seq bulked segregant analysis combined with KASP genotyping rapidly identified *PmCH7087* as responsible for powdery mildew resistance in wheat. Plant Genome 14:1–13. 10.1002/tpg2.20120 10.1002/tpg2.20120 PMC12807311 34309200

[CR51] Zong C, Yue Y, Shao G et al (2010) Effects of multifoliolate compound leaf on photosynthetic characteristics and yield of soybean. Soybean Sci 29:627–633

