# Supplementary material for: QTL mapping and BSR-seq revealed loci and candidate genes associated with the sporadic multifoliolate phenotype in soybean (Glycine max)
Source: Theor Appl Genet. 2024 Nov 8;137(12):262. doi: 10.1007/s00122-024-04765-z (PMC11543727; doi:10.1007/s00122-024-04765-z)
Supplement: Supplementary file 1 — Supplementary file1 (DOCX 15 KB) [file 122_2024_4765_MOESM1_ESM.docx]

**Table S1**. Phenotypic variation and heritability of the multifoliolate phenotype in the recombinant inbred line (RIL) population of C08 x W05 with three biological replicates.

| **Replicates** |  | **No. of plants with multifoliolate leaves in C08** | **No. of plants with multifoliolate leaves in W05** | **No. of plants with multifoliolate leaves within each RIL** | **Mean ± SD** | **CV** | ***H^2^*** |
| --- | --- | --- | --- | --- | --- | --- | --- |
| Rep1 |  | 0 | 3 | 0-4 | 0.5504±1.0228 | 0.53813062 | 0.6816956 |
| Rep2 |  | 0 | 4 | 0-9 | 1.3005±2.0961 | 0.62043796 |  |
| Rep3 |  | 0 | 7 | 0-20 | 3.7143±4.7968 | 0.77432872 |  |

CV, Coefficient of variation; *H^2^*, broad-sense heritability; SD, Standard deviation.
