# Supplementary material for: QTL mapping and BSR-seq revealed loci and candidate genes associated with the sporadic multifoliolate phenotype in soybean (Glycine max)
Source: Theor Appl Genet. 2024 Nov 8;137(12):262. doi: 10.1007/s00122-024-04765-z (PMC11543727; doi:10.1007/s00122-024-04765-z)
Supplement: Supplementary file 4 — Supplementary file4 (DOCX 18 KB) [file 122_2024_4765_MOESM4_ESM.docx]

**Table S4.** Significant genetic regions based on ΔSNP indices between V0-MUL and V0-TRI.

| [**Chromosome**](javascript:;) | **Start position** | **End position** | **Size (Mb)** | **Number of genes** |
| --- | --- | --- | --- | --- |
| Gm02 | 16190000 | 17330000 | 1.14 | 34 |
| Gm02 | 19630000 | 21100000 | 1.47 | 5 |
| Gm02 | 22450000 | 23600000 | 1.15 | 9 |
| Gm02 | 23610000 | 25000000 | 1.39 | 3 |
| Gm02 | 26650000 | 27770000 | 1.12 | 14 |
| Gm02 | 30850000 | 31900000 | 1.05 | 14 |
| Gm02 | 32820000 | 33960000 | 1.14 | 24 |
| Gm02 | 34440000 | 35800000 | 1.36 | 12 |
| Gm06 | 17560000 | 19470000 | 1.91 | 82 |
| Gm06 | 24630000 | 26250000 | 1.62 | 2 |
| Gm06 | 26550000 | 27550000 | 1.00 | 4 |
| Gm06 | 28020000 | 29120000 | 1.10 | 9 |
| Gm06 | 30430000 | 32160000 | 1.73 | 6 |
| Gm06 | 32820000 | 33880000 | 1.06 | 5 |
| Gm09 | 26530000 | 27560000 | 1.03 | 2 |
| Gm14 | 37070000 | 38330000 | 1.26 | 5 |
| Gm19 | 24180000 | 26460000 | 2.28 | 19 |
| Gm20 | 26640000 | 27660000 | 1.02 | 15 |
| Gm08 | 6560000 | 9150000 | 2.59 | 324 |
| Gm08 | 30860000 | 32690000 | 1.83 | 9 |
| Gm08 | 33020000 | 34070000 | 1.05 | 10 |
| Gm12 | 29690000 | 30800000 | 1.11 | 7 |
| Gm17 | 29190000 | 30360000 | 1.17 | 7 |

MUL, high-multifoliolate frequency bulk; TRI, low-multifoliolate frequency bulk; V0, shoot apical bud from the true leaf stage.
