# Supplementary material for: QTL mapping and BSR-seq revealed loci and candidate genes associated with the sporadic multifoliolate phenotype in soybean (Glycine max)
Source: Theor Appl Genet. 2024 Nov 8;137(12):262. doi: 10.1007/s00122-024-04765-z (PMC11543727; doi:10.1007/s00122-024-04765-z)
Supplement: Supplementary file 5 — Supplementary file5 (DOCX 17 KB) [file 122_2024_4765_MOESM5_ESM.docx]

**Table S5.** Significant genetic regions based on ΔSNP indices between V1-MUL and V1-TRI.

| [**Chromosome**](javascript:;) | **Start position** | **End position** | **Size (Mb)** | **Number of genes** |
| --- | --- | --- | --- | --- |
| Gm02 | 20500000 | 22430000 | 1.93 | 6 |
| Gm02 | 25750000 | 27020000 | 1.27 | 10 |
| Gm02 | 34610000 | 35900000 | 1.29 | 12 |
| Gm04 | 28670000 | 30180000 | 1.51 | 13 |
| Gm04 | 33460000 | 34500000 | 1.04 | 7 |
| Gm04 | 35890000 | 36950000 | 1.06 | 5 |
| Gm20 | 14760000 | 16660000 | 1.90 | 3 |
| Gm20 | 27830000 | 28890000 | 1.06 | 9 |
| Gm08 | 30860000 | 32680000 | 1.82 | 9 |
| Gm08 | 33020000 | 34070000 | 1.05 | 10 |
| Gm08 | 36070000 | 37160000 | 1.09 | 27 |
| Gm12 | 11650000 | 12720000 | 1.07 | 23 |
| Gm12 | 24570000 | 26120000 | 1.55 | 14 |

MUL, high-multifoliolate frequency bulk; TRI, low-multifoliolate frequency bulk; V1, leaf tissue from the first compound leaf.
