# Supplementary material for: QTL mapping and BSR-seq revealed loci and candidate genes associated with the sporadic multifoliolate phenotype in soybean (Glycine max)
Source: Theor Appl Genet. 2024 Nov 8;137(12):262. doi: 10.1007/s00122-024-04765-z (PMC11543727; doi:10.1007/s00122-024-04765-z)
Supplement: Supplementary file 8 — Supplementary file8 (DOCX 6836 KB) [file 122_2024_4765_MOESM8_ESM.docx]

**Fig. S1**. Pearson correlation coefficients of the numbers of lines with multifoliolate seedlings among three biological replicates. Blue circles represent positive correlations. The size and color intensity of each circle are proportional to the absolute value of the correlation coefficient. The values shown are the correlation coefficients between different replicates. ^***^, *P*<0.001.

**Fig. S2.** Pearson correlation coefficients between the multifoliolate phenotype and various yield-related traits. Blue circles represent positive correlations. The size and color intensity of each circle are proportional to the absolute value of the correlation coefficient. The values shown are the correlation coefficients between different traits. ^***^, *P*<0.001; ^*^, *P*<0.05. MF, the number of multifoliolate seedlings; S100, 100-seed weight.


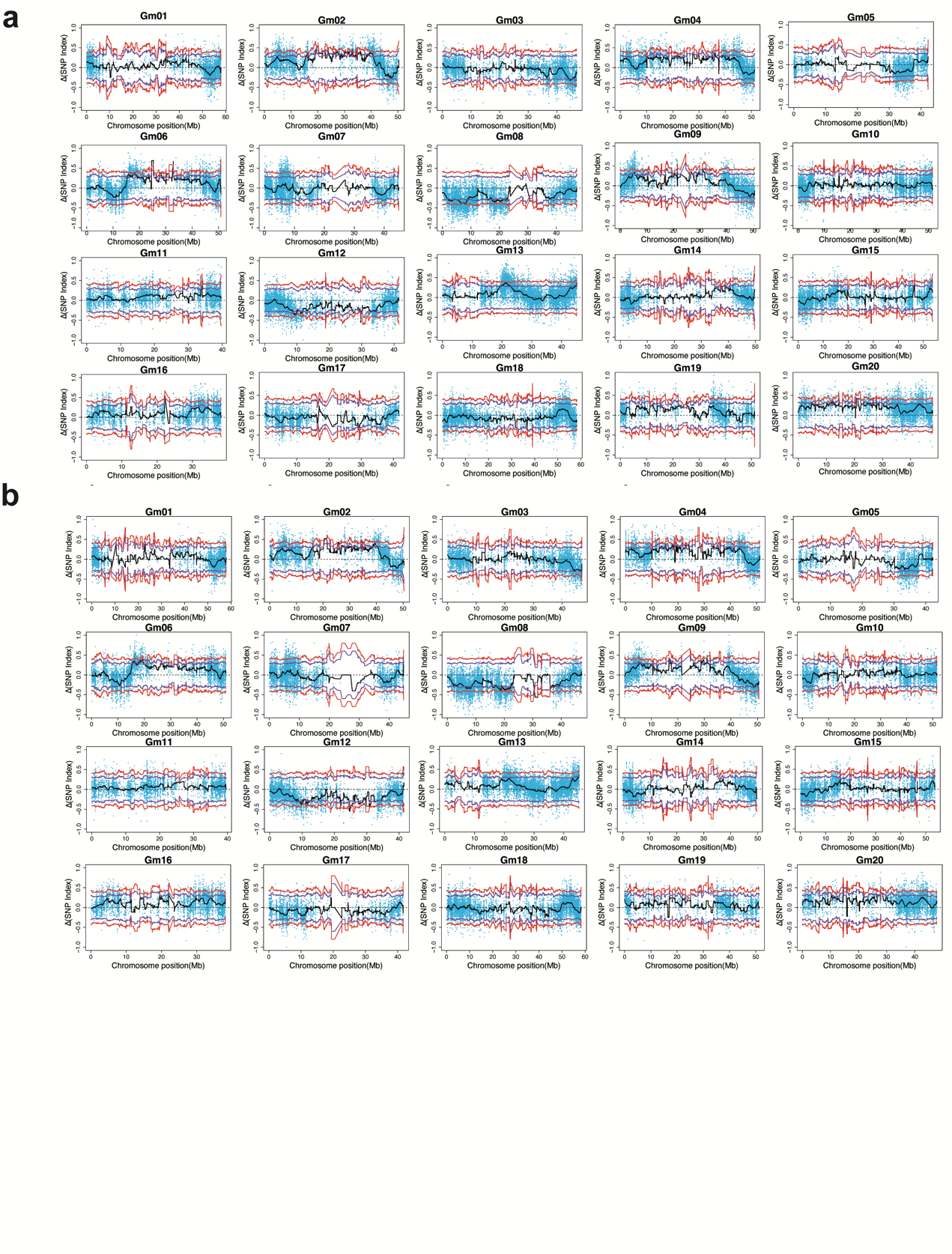


**Fig. S3**. Bulked segregant RNA sequencing (BSR-seq) analyses of the multifoliolate phenotype in soybean. (a) Δ(SNP-index) plot on 20 chromosomes for the comparison between V0-MUL and V0-TRI. (b) Δ(SNP-index) plot on 20 chromosomes for the comparison between V1-MUL and V1-TRI. Red lines indicate the 99% confidence intervals and purple lines indicate the 95% confidence interval. V0, first fully opened true leaf stage; V1, first fully opened compound leaf stage; MUL, high-multifoliolate frequency bulk; TRI, low-multifoliolate frequency bulk.
